# Opt-out as an acceptable method of obtaining consent in medical research: a short report

**DOI:** 10.1186/1471-2288-11-40

**Published:** 2011-04-06

**Authors:** Akke Vellinga, Martin Cormican, Belinda Hanahoe, Kathleen Bennett, Andrew W Murphy

**Affiliations:** 1 Discipline of General Practice, School of Medicine, NUI Galway, Ireland; 2 Discipline of Bacteriology, School of Medicine, NUI Galway, Ireland; 3 Department of Medical Microbiology, University Hospital, Galway, Ireland; 4 Department of Pharmacology & Therapeutics, Trinity Centre for Health Sciences, Dublin, Ireland

## Abstract

**Background:**

A prospective cohort study was set up to investigate a possible association between antibiotic prescribing and antibiotic resistance of *E. coli *urinary tract infection in the community. Participation of patients with urinary tract infection was obtained through an opt-out methodology. This short paper reports on the acceptability of the opt-out recruitment approach.

**Methods:**

Participating practices (22) were requested to send a urine sample from all patients presenting with symptoms of urinary tract infection. Upon receipt of the sample in the laboratory, a letter explaining the study, an opt-out form and a freepost envelope were sent to all adult patients. A website with additional information and including an 'opt-out' button was set up for the study.

**Results:**

A total of 1362 urine samples were submitted by the 22 participating practices representing 1178 adult patients of whom 193 actively responded to the letter: 142 opted out by letter, 15 through the website, 2 by phone and 12 sent the letter back without indication, making a total of 171 patients or 14.5% opt-out; the remaining 22 patients (1.9%) explicitly opted in. The total group consisted of 80% women and the mean age was 50.9 years (sd 20.8). No significant differences were found between patients who participated and those who opted out in terms of age, gender or whether the urine sample was positive or not.

**Conclusions:**

Overall the opt-out method was well received and participation in the study reached 85.5%. The low number of complaints (2) indicates that this is a generally acceptable method of patient recruitment. The 14.5% opt-out shows that it effectively empowers patients to decline participation. The similarity between patients opting out and the rest of the patients is reassuring for extrapolation of the results of the study.

## Background

The gold standard with respect to ethical recruitment of participants in research is explicit written consent, although various studies have shown that most patients do not have a preference for active consent [1]. An Irish study on public perceptions of biomedical research found that the public is generally aware of and committed to making a contribution to research and related activities in the healthcare system for their benefit and for the benefit of future patients [2]. In our study on management of urinary tract infection (UTI) in general practice, it was important to get a representative sample of patients. Concern was raised that an opt-in method for recruitment could cause bias as this approach is time consuming for the general practitioner (GP) which could impact on the participation of the GP and the patient. This short paper gives an overview of the application of an opt-out recruitment approach and its acceptability for consideration in other health-related studies.

### Overview of the justification to support opt-out consent

Active consent or opt-in has been shown to limit participation [3] and introduce bias into studies [4]. If consent is considered an indication of willingness rather than refusal and if risks for the participants are very low, an opt-out arrangement or passive consent is generally the most efficient procedure without violating the option of providing choice [5]. This methodology will most likely result in a more representative population reflecting the real life situation [6].

A limited number of studies explicitly researched possible objections to the opt-out system and not only found this method to be generally accepted but additionally identified patients' appreciation of participation in research [7,8].

Good methodology should respect personal autonomy by providing the necessary information to make an informed decision and include safeguards to protect privacy [9-11]. The challenge in applying an opt-out methodology is to provide easily accessible information to all patients to facilitate informed consent without interfering with the medical consultation. This principle was applied to our study on antimicrobial resistance and prescribing in adults with urinary tract infections.

## Methods

A prospective cohort study was set up to describe the management of UTI in Irish general practice as well as to investigate a possible association between antimicrobial prescribing and resistance of *E. coli *isolated from patients with urinary tract infection.

Following extensive dialogue with the Research Ethics Committee of the Irish College of General Practitioners, approval for an opt-out consent method was given. The study received ethical approval for the use of an opt-out methodology based on the low risk to the patient and the potential benefit for the patient of adequate management of UTI based on unbiased information [5,12]. In this interpretation consent is an indication of willingness rather than refusal and informed consent is obtained by generally accessible information as well as easy modes to opt out [11].

Only one laboratory provides microbiological services in this region. After a retrospective analysis of laboratory data from all practices submitting to this laboratory [13], a list of practices was generated based on the number of positive urine samples in 2007. In an effort to limit the workload of a potential increase in the number of samples due to the study and in consultation with the laboratory, the highest ranking practices were selected. The top 25 practices were invited and 22 agreed to participate in the study, two practices did not have computerised records and one practice declined. Practices were located in rural as well as urban locations with a variety of patient populations. Practices had different patient (age and gender) profiles as well as different proportions of private and medical card patients. At the time of the study about 30% of the population was eligible for a medical card. Medical card eligibility is determined by income as well as age (all pensioners over the age of 70 years are eligible). Medical card patients have free medical care and medication [14]. The participating general practices were considered representative of all Irish general practices.

All practices received posters informing patients of the study to display in the waiting room, as well as little reminder cards for the consultation rooms. Participating practices were requested to send a urine sample from all patients presenting with symptoms suggestive of urinary tract infection. Upon receipt of the sample in the laboratory, a letter explaining the study (supplementary file), an opt-out form and a freepost envelope were sent to all adult patients who supplied a urine sample.

In the letter patients were informed of the objectives of the study and permission for the researcher to look at their GP records was requested. Additionally, it was explained that they were free to opt out of participation in the study by filling out the included opt-out form, by phone or through the website.

A dedicated website was set up with detailed information on the study as well as on the problem of antimicrobial resistance in general http://www.antibiotics.nuigalway.ie. The index page included an 'opt-out' button which linked to a form that could be filled out by the patient. The website was clearly laid out to avoid confusion and ensure easy opt-out. Regular updates on the study, as well as study results were added to the website when available.

## Results

A total of 1362 urine samples were submitted by the 22 participating practices during the study period. The samples were from 1178 adult patients. The 22 practices sent in between 15 and 115 samples.

**Figure 1:**
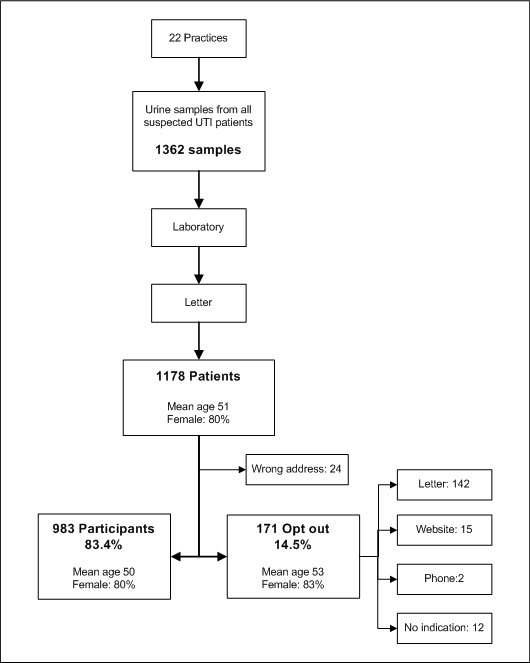
**Flow chart of the participation and opt-out of patients in the study**.

In total 193 patients actively responded to the letter: 142 opted out by letter, 15 through the website, 2 by phone and 12 sent the letter back without indication, making a total of 171 patients (14.5%) who opted out; the remaining 22 patients (1.9%) explicitly opted in (Figure 1). The letters of 24 patients had a wrong address and were returned.

Two patients expressed concerns regarding the use of the opt-out method. Both questioned the way their address was obtained and whether this interfered with the confidentiality of their patient data. An individual response to these concerns was sent to their GP with a request to forward this to the patient. No further concerns were expressed.

Patients consisted of 941 women (79.9%) and 237 men (20.1%). Their mean age was 50.9 years (sd 20.8) and the median age was 47 years. Patients who opted out were slightly older (52.8 vs 50.4 years) and the percentage of females was slightly higher (83% vs 79.5%) but these differences were not statistically significant. Patients who opted out through the website were significantly younger than those who used the letter (non-parametric, 53.5 vs 38.7 years, p < 0.05).

A significant isolate (pure culture at greater than 10^5 ^colony forming units/ml) was identified from the urine sample of 402 (34.1%) patients. Patients with a positive culture were no more likely to opt out compared to those with a negative culture.

## Discussion and Conclusions

Overall the opt-out method was well received by both general practitioner and patients and achieved a high level of participation in the study at 83.4%. The low number of complaints indicates that this is a generally acceptable method of patient recruitment. The 14.5% opt-out of patients shows that the process effectively empowered patients to decline participation. The high similarity between patients opting out and the participating patients with respect to age, gender and isolation of a positive culture is reassuring for extrapolation of the results of the study. However, as no other potentially important variables were available about the patients who opted out, it cannot be ruled out that other factors were of importance for participation in the study. Similarly, even though every effort was made to inform patients of the study, it cannot be guaranteed that all patients received this information through the different media offered by us.

Our findings are in line with other studies which have shown that an opt-out methodology is generally well accepted and will result in high participation rates [7,8,15]. A recent Cochrane review looked at ways to increase recruitment into clinical studies and also identified opt- out as a possible method [16]. The lack of further involvement in the study by participants and general practitioners, acknowledged in the ethical approval given to the study, favours this type of recruitment which might be less applicable for studies with more involvement or risk. For non-interventional, low-risk studies in which rigorous measure to inform patients and protect patient confidentiality are in place, recruitment by opt-out is an easy and acceptable methodology for patients, GPs and researchers. As earlier stated by Junghans et al. [4], the opt-out approach should be the default recruitment strategy for studies with low risk to participants.

## Competing interests

The authors declare that they have no competing interests.

## Funding

This research project was funded by a grant from the Health Research Board, Ireland

## Authors' contributions

AV set up and coordinated the study, analysed the results and drafted the manuscript. MC and AWM conceived of the study and critically revised the manuscript. BH acquired the data for the study and approved the final manuscript. KB has been involved in the conception of the study, acquisition of data and has approved the final manuscript. All authors read and approved the final draft.

## Pre-publication history

The pre-publication history for this paper can be accessed here:

http://www.biomedcentral.com/1471-2288/11/40/prepub
